# Pollinators and herbivores interactively shape selection on strawberry defence and attraction

**DOI:** 10.1002/evl3.262

**Published:** 2021-11-14

**Authors:** Paul A. Egan, Anne Muola, Amy L. Parachnowitsch, Johan A. Stenberg

**Affiliations:** ^1^ Department of Plant Protection Biology Swedish University of Agricultural Sciences Alnarp SE‐23053 Sweden; ^2^ Biodiversity Unit University of Turku Turku 20014 Finland; ^3^ Department of Biology University of New Brunswick Fredericton NB E3B 5A3 Canada; ^4^ Department of Plant Ecology and Evolution, Evolutionary Biology Centre Uppsala University Uppsala SE‐75236 Sweden

**Keywords:** Conflicting selection, diffuse selection, eco‐evolutionary dynamics, *Fragaria vesca*, multispecies interactions, plant‐herbivore‐pollinator

## Abstract

Tripartite interactions between plants, herbivores, and pollinators hold fitness consequences for most angiosperms. However, little is known on how plants evolve in response—and in particular what the net selective outcomes are for traits of shared relevance to pollinators and herbivores. In this study, we manipulated herbivory (“presence” and “absence” treatments) and pollination (“open” and “hand pollination” treatments) in a full factorial common‐garden experiment with woodland strawberry (*Fragaria vesca* L.). This design allowed us to quantify the relative importance and interactive effects of herbivore‐ and pollinator‐mediated selection on nine traits related to plant defence and attraction. Our results showed that pollinators imposed stronger selection than herbivores on traits related to both direct and indirect (i.e., tritrophic) defence. However, conflicting selection was imposed on inflorescence density: a trait that appears to be shared by herbivores and pollinators as a host plant signal. However, in all cases, selection imposed by one agent depended largely on the presence or ecological effect of the other, suggesting that dynamic patterns of selection could be a common outcome of these interactions in natural populations. As a whole, our findings highlight the significance of plant‐herbivore‐pollinator interactions as potential drivers of evolutionary change, and reveal that pollinators likely play an underappreciated role as selective agents on direct and in direct plant defence.

**Impact Summary**

This study advances understanding of plant‐herbivore‐pollinator interactions in revealing how these interactions can influence the phenotypic evolution of plant defence and attractive traits.

A full‐factorial manipulation of pollination and herbivory (in a common‐garden experiment approach with woodland strawberry) enabled our study to quantify and compare the relative strength, direction, and dynamics of selection by these agents.

Herbivores have long been considered the primary drivers of defence trait evolution in plant populations. We offer a novel counter to this preconception, and provide the first empirical support demonstrating the importance of pollinators as selective agents on direct and indirect defence traits, which here even surpassed that of herbivores.

We furthermore revealed that the net selective pressures arising from these interactions were highly dynamic, and depended largely on the presence or ecological effects of the other agent. These findings are important given there remains a lack of studies that simultaneously quantify pollinator‐ and herbivore‐mediated selection on the same trait.

As a whole, our study highlights the possible evolutionary trajectories of defence and attractive traits in natural populations, and how pollinator and herbivore selective pressures are expected to interactively shape this evolution.

To maximize reproductive success, most angiosperms must attract pollinating mutualists but evade herbivore antagonists (Strauss 1997; Lucas‐Barbosa 2016; Kessler and Chautá 2020). The plant traits that mediate these interactions are often interlinked (Theis et al. 2007; González‐Teuber and Heil 2009; Galen et al. 2011; Kessler et al. 2019; Rusman et al. 2019), which can result in fitness trade‐offs for plants (Herrera et al. 2002). For instance, pollinators often preferentially forage on larger, more apparent flowers and inflorescence displays (Conner and Rush 1996; Parachnowitsch and Kessler 2010). Yet greater visual or olfactory apparency can incur fitness costs when these traits are co‐opted by herbivores as “shared signals” to the host plant (Halitschke et al. 2008; Sletvold and Grindeland 2008; Theis and Adler 2012; Knauer and Schiestl 2017; Santangelo et al. 2019). Plant‐herbivore interactions can likewise bear consequences for pollinator‐mediated fitness (Kessler et al. 2011; Muola et al. 2017). The presence and action of herbivores, and in particular their damage to leaves (folivory) and flowers (florivory), can deter pollinators via a range of mechanisms (Jacobsen and Raguso 2018; Moreira et al. 2019; Haas and Lortie 2020). Pollinator deterrence may be based on visual, olfactory, or gustatory cues, which can include altered floral aesthetics (McCall and Irwin 2006), release of herbivore‐induced plant volatiles (Kessler and Chautá 2020), or the induction of unpalatable defensive compounds in floral nectar (Adler et al. 2006). Given the multiple routes by which these ecological effects can manifest for plants, the net outcomes for fitness are expected to be highly context specific (e.g., Gegear et al. 2007).

Yet despite growing appreciation of the ecology of plant‐herbivore‐pollinator interactions, it remains poorly understood how these interactions ultimately affect phenotypic selection on traits at the microevolutionary scale. The high degree of ecological linkage inherent in these interactions suggests that pollinator and herbivore selection on traits may often be conflicting (i.e., exerted in opposing directions) and diffuse or nonadditive in nature (i.e., dependent on the presence or ecological effects of the other selective agent) (Ashman et al. 2004; Strauss et al. 2005; Sletvold et al. 2015; TerHorst et al. 2015; Knauer and Schiestl 2017; Ramos and Schiestl 2019; Sletvold 2019). Conflicting selection by pollinators and herbivores has been found on flowering phenology in an orchid system (Sletvold et al. 2015), and on a floral scent compound in *Brassica rapa* L. (Knauer and Schiestl 2017). These studies reinforce the notion that pollinator‐ and herbivore‐mediated selection may often be imposed nonindependently, and result in complex patterns of net selection on traits.

Although herbivory is believed to generally constrain floral evolution (Ashman 2002; Johnson et al. 2015; Jogesh et al. 2017; Ramos and Schiestl 2019; Santangelo et al. 2019), a recent meta‐analysis by Caruso et al. (2019) revealed a general lack of studies that simultaneously quantify pollinator‐ and herbivore‐mediated selection on the same trait. Further studies are required to examine and compare the relative importance and dynamics of pollinator and herbivore selection on traits that link pollination and herbivory. Beyond floral traits, these should also include traits that are important for direct defence (e.g., chemical compounds; especially if they also occur in floral rewards) and indirect defence mediated by the natural enemies of herbivores (e.g., predatory and parasitoid arthropods). Although few studies have examined the potential for pollinators to select on defence traits related to herbivory (Kessler and Halitschke 2009; Egan 2015; Ramos and Schiestl 2019), such selection may in fact be commonplace in plants (Egan et al. 2016), especially as an adaptation to mitigate herbivore deterrence of pollinators.

In this study, we manipulated pollination and herbivory in a common‐garden experiment with woodland strawberry (*Fragaria vesca* L.). We examined phenotypic selection on several chemical traits previously identified as markers of direct and indirect defence for this species (see below and Weber et al. 2020a, 2020b). In addition, we also examined selection on several floral and vegetative morphological traits potentially important for attraction of pollinators and herbivores, including flower frost tolerance. Although abiotic stress may commonly drive selection on this latter trait (Agrawal et al. 2004), few studies have examined if frost‐damaged flowers could impact pollinator interactions (Pardee et al. 2018) or pollinator‐mediated selection. We tested the hypotheses that (1) pollinators and herbivores positively select on defence‐related traits—traits that in our system are expected to aid against the direct and pollinator‐mediated costs of herbivory; (2) pollinators and herbivores impose conflicting selection on plant attractive traits—as potential shared host‐selection cues used by both agents; and (3) that the above selection regimes are diffuse—that, that pollinator‐mediated selection is modified (in strength, and possibly direction) when herbivory is manipulated, and vice versa. Investigations of this kind can thereby provide greater insight into the eco‐evolutionary dynamics of plant‐herbivore‐pollinator interactions.

## Methods

### STUDY SYSTEM

*Fragaria vesca* is native to Eurasia (ssp. *vesca*) and North America (ssp. *americana*; ssp. *bracteata*) where it grows in diverse conditions from boreal to Mediterranean climates and in habitats ranging from disturbed forest margins to open grasslands (Maliníková et al. 2013). The species is perennial and predominantly hermaphrodite and self‐compatible, but often still largely benefits from cross‐pollination (Liston et al. 2014). Hoverflies and bumblebees, and to a lesser extent solitary bees, were observed as the most frequent flower visitors throughout the conduct of this experiment (pers. obs.). In Northern Europe, *F. vesca* is frequently attacked by several insect herbivores, but especially the strawberry leaf beetle *Galerucella tenella* L. (Coleoptera: Chrysomelidae). This oligophagous herbivore feeds on leaves and flowers of several Rosaceae plants (Stenberg and Axelsson 2008). We previously demonstrated that damage by the strawberry leaf beetle can reduce *F. vesca* fitness both directly and indirectly, by causing pollinator limitation (Muola et al. 2017; Muola and Stenberg 2018).

### COMMON GARDEN

A common‐garden experiment was initiated in which pollination and herbivory were manipulated to examine phenotypic selection on nine plant attractive and defence‐related traits in *F. vesca*. Details of the sourcing and propagation of 100 plant genotypes from wild Swedish populations are provided in Appendix S1. The common garden consisted of 400 plants grown across four blocks spaced 1 m apart. Due to a small amount of overwintering mortality, only 81 genotypes that had complete replication were subsequently used in this study (listed in Table S1). The experiment employed a split‐plot design in which two pollination treatments (open vs. hand pollination) were applied within blocks, and two herbivory treatments (herbivore addition vs. removal) were applied in a spatially alternating sequence across blocks, to afford a total of four unique treatment combinations. This experimental design thus had the power to reveal the relative strength and direction of selection imposed by pollinators and herbivores on traits, and how dynamic these selection patterns were when the influence of the other agent was controlled for.

### POLLINATION AND HERBIVORY TREATMENTS

For the pollination treatments, all flowers on a plant were either exposed to ambient pollination conditions (open pollination) or received supplemental hand pollinations every about 5 days for the full flowering period. Only three plant genotypes were used as pollen donors to control for pollen quality effects. Pollen was applied using a fine‐hair paintbrush ensuring that no hand contact was made with plants during application. Herbivory was manipulated through the addition or removal of an ecologically important herbivore: the strawberry leaf beetle, *G. tenella*. The plantation in which all treatments were applied was fenced with a 2‐m fine mesh to exclude herbivory from small or large browsing vertebrates, including deer and digging rodents. For the “herbivore addition” treatment, several hundred adult individuals of the strawberry leaf beetle were collected in early May soon after their emergence on their main host plant meadowsweet (*Filipendula ulmaria* [L.] Maxim.) (Stenberg and Axelsson 2008). Further details of these collection localities are provided in Weber et al. (2020b). The beetles were released onto plants at a density of 0.4 individuals per plant. This was the highest density permitted by numbers available from the collection localities, but which was nonetheless sufficient to cause moderate to high damage severity in these blocks (average no. damaged leaves per plant = 29.5% ± 14.7 SD). Adult and larval feeding damage were apparent within 1 and 5 weeks of release, respectively. Most feeding damage in the “herbivore addition” treatment was hence caused by the strawberry leaf beetle. However, damage from larvae of two species of leaf‐ and flower‐feeding Lepidoptera—*Cnephasia asseclana* Denis and Schiffermüller (Tortricidae) and *Ceramica pisi* L. (Noctuidae)—was also observed on experimental plants. The former is a pest of cultivated strawberry (Sigsgaard et al. 2014), whereas the latter is a generalist moth that feeds on genera including *Rubus* and *Salix* (Robinson et al. 2010). As a common practice for the experimental removal of insect herbivores (Siemann et al. 2008), the “herbivore removal” treatment was made through application of low doses of an insecticide. For this, we employed foliar applications of Calypso (Bayer CropScience); a systemic insecticide based on the active substance thiacloprid. All manufacturers’ recommendations were followed concerning dosage concentration and frequency of application. Three rounds of application were made every about 4 weeks between early May and mid‐July. Spraying took place when conditions were dry and windless to avoid cross‐contamination, and at the same time plants in the “herbivore addition” treatment were sprayed with the equivalent volume of water. Virtually no damage to plants was observed in the “herbivore removal” treatment (average no. damaged leaves per plant = 0.1% ± 1.2 SD).

### TRAIT MEASUREMENTS

The plant genotypes used in this study were previously shown to harbor high genetic variation in direct and indirect defence against the strawberry leaf beetle (Weber et al. 2020a, 2020b). Variation in direct and indirect defence was evidenced by the differential performance and preference of the herbivore and its specialist endoparasitoid (*Asecodes parviclava* Thompson [Hymenoptera: Eulophidae]) on diets of varying genotypic composition. Several primary and secondary compounds were ultimately identified as markers associated with either direct or indirect defence (Weber et al. 2020a). We included the five compounds most strongly associated with defence: two carbohydrates (dehydroascorbic acid and myo‐inositol) and three phenolics (catechin, dihydroxybenzoic acid, and shikimic acid). The former carbohydrate was associated with successful parasitism of the herbivore host (by possibly facilitating parasitoid development), and the latter with mortality of the host before parasitism was complete. The three phenolics were associated with reduced growth rate of the herbivore (measured in bioassays not involving parasitism), and in the case of dihydroxybenzoic acid also with parasitism success. Details of leaf sampling and GC/TOF‐MS metabolomic profiling are presented in Weber et al. (2020a), which was conducted concurrently for the same plants used in this study. Owing to capacity limitations experienced with metabolomic profiling, defence‐related traits could only be quantified for 27 of the 81 genotypes. However, selection of these 27 genotypes was made such that the full spectrum of genetic variation in direct and indirect defence was still represented (Table S1).

The four attractive traits examined were plant size, total flower number, flower frost tolerance, and inflorescence density, as in Egan et al. (2018). Plant size was quantified as the volume of a partial sphere (in dm^3^) from measurements of plant length and width. Given their prior establishment for 1 year, plants were generally expected to have obtained their maximal size by the time traits were measured (mean plant size = 7.5 dm^3^ ± 3.0 SD). The latter three traits were based on flower counts (mean number of flowers per plant = 73.9 ± 30.3 SD). Frost‐damaged flowers showed a complete blackening of the receptacle at the center of the flower, and were hence hypothesized to negatively impact floral attractiveness to pollinators and florivores—possibly due to a lack of material for consumption (i.e., nectar or pollen rewards for pollinators; floral tissue for florivores). No visitation on frost‐damaged flowers was observed during the experiment, although visitation to nondamaged flowers on the same plant still occurred. Inflorescence density was quantified as the number of flowers per dm^3^. Conceptually, this trait differs from flower number in that it describes a different feature of relevance to pollinator or florivore attraction; that plants with many flowers could have these quite dispersed in the inflorescence, and vice versa. Bumblebees were also frequently seen to “walk” between flowers when the inflorescence was dense (pers. obs.), suggesting that this trait had the potential to be selected for independently of flower number. Phenotypic correlations between most of these traits were low (maximum *r* ^2^ = 0.08; see Egan et al. 2018) but were nonetheless accounted for through use of multiple regression analyses as described below.

### FITNESS MEASUREMENT

Plant fitness was measured as total seed output per plant. For this, berries were picked fresh as they ripened on plants up until most fruiting had finished by mid‐July. After this time, berries were allowed to dry on the plant before a final picking in early August. Although birds were not excluded from the common garden, we did not observe any bird frugivory. All berries were dried in an oven at 80°C for 1.5 days. To estimate the total number of fertilized seeds per plant from dry berry weight, we established two regression equations; one for berries that were picked fresh (*y* = 0.9937*x* – 14.257, *R* ^2^ > 0.99), and one for berries that were picked dry (*y* = 1.0017*x* – 14.423, *R* ^2^ > 0.99). Berries were randomly selected across all genotypes and treatments to establish these equations. Only fertilized seeds were counted, which are easily visually differentiated from nonfertilized seeds (Thompson 1971). Owing to their much‐reduced size, nonfertilized seeds made only a negligible contribution to dry berry weight (data not shown).

### PHENOTYPIC SELECTION ANALYSIS

To quantify phenotypic selection on traits, multiple regression was used to provide estimates of selection gradients, whereas individual univariate models were used to provide estimates of selection differentials. Selection gradients (β) describe the strength of unique or direct selection acting on a trait (after controlling for inter‐trait correlations), whereas selection differentials (S) describe “total” selection (Lande and Arnold 1983). Within each of the four treatment combinations, fitness was first relativized by dividing seed number by its mean, and traits were standardized by their standard deviation (Lande and Arnold 1983). For the analysis of β, a multiple regression model was fitted in which relative fitness was regressed on standardized traits. We also included in the model the two‐level factors of “pollination” (open, hand pollination) and “herbivory” (addition, removal), and their interaction with all traits and each other. In this way, estimates of β were output for all traits in all four treatment combinations. Following Sletvold (2019), the calculated difference in fitness‐trait slopes (β) between treatment combinations provided estimates of pollinator‐mediated selection (both in the presence and absence of herbivory), herbivore‐mediated selection (with and without pollinator limitation), and combined pollinator‐ and herbivore‐mediated selection. Table S2 details the exact treatment combinations used to calculate the estimates, and ANOVA outputs from the main model are included in Table S3. The “emtrends” function of R package emmeans (Lenth et al. 2018) was used to calculate these differences in β, and to test whether the result differed significantly from zero (after adjusting *P*‐values for multiple comparisons via Benjamini‐Hochberg correction). Although the multiple regression included only 27 genotypes for which complete trait data were available, we still considered these inferences to be robust given that (1) the full spectrum of genetic variation in defence was represented (see above and Table S1); and (2) no qualitative differences were observed in univariate regressions regardless of whether some (*n* = 27) or all (*n* = 81) genotypes were used (Table S4). For the analysis of S, univariate regressions were fitted for each trait individually. Only data from the “open pollination/herbivore present” treatment combination were used for the estimation of S, as this treatment combination is typically considered most representative of natural population conditions (Sletvold 2019).

## Results

**Figure 1:**
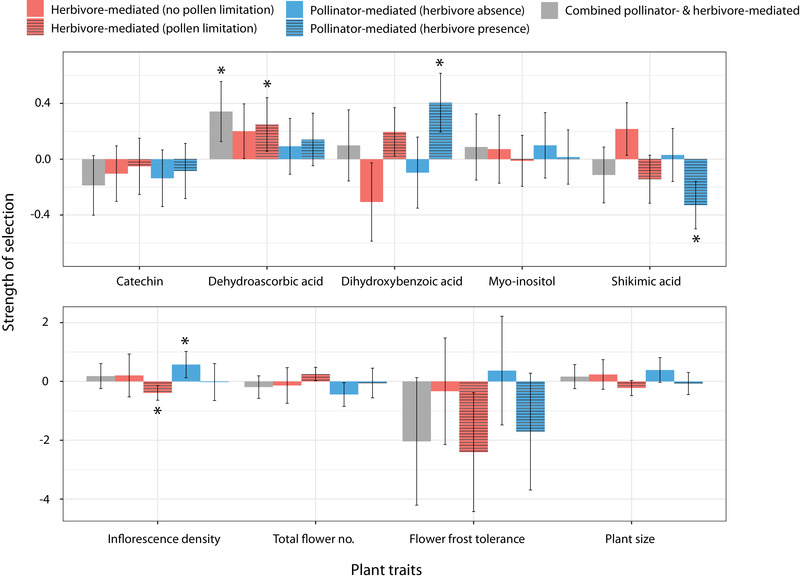
Relative direction and strength of phenotypic selection mediated by pollinators and herbivores on defence‐related traits (top) and plant attractive traits (bottom) in woodland strawberry (*Fragaria vesca*). Pollinator‐ and herbivore‐mediated selection was quantified both under control and manipulated conditions to reveal whether selection was diffuse (context‐dependent on the other agent) and/or conflicting (exerted in opposing directions). Presented are selection gradient (β) coefficients and their associated confidence intervals. Asterisks indicate that selection is significantly different from zero, following adjustment of *P*‐values for multiple comparisons (see *Methods*).

Of the nine traits examined in this study, pollinators were implicated as agents of selection on four (three defence‐related and one attractive), and herbivores on two (one defence‐related and one attractive) (Fig. 1). Pollinators generally imposed stronger selection pressures than herbivores, and only pollinator‐mediated selection appeared to constitute an important component of “total” selection overall (Table S4)—that is, for dihydroxybenzoic acid (net positive selection) and shikimic acid (net negative selection). Conflicting selection—where pollinators and herbivores exerted selection in opposing directions—was observed for inflorescence density, for which these agents imposed positive and negative selection, respectively. However, each of the above selection regimes was diffuse—meaning that the strength of selection by one agent was context depended upon (or modified by) the presence or ecological effects of the other (Fig. 1). In particular, selection by pollinators or herbivores was only detected in the presence or absence of the other agent but never in both states.

## Discussion

### SELECTION ON DIRECT DEFENCE

Herbivores have long been considered the primary drivers of defence trait evolution in plant populations (Johnson et al. 2015). However, in line with our predictions, pollinators imposed positive directional selection on direct defence‐related traits in one instance (for dihydroxybenzoic acid), although negative directional selection was also surprisingly apparent (for shikimic acid) (Fig. 1). Dihydroxybenzoic acid is a phenolic that is strongly associated with resistance against the strawberry leaf beetle (Weber et al. 2020a). Given that pollinators were previously found to avoid damaged flowers of woodland strawberry (Muola et al. 2017), it appears logical that pollinator selection on this defence compound was exerted only in the presence of herbivores, and not in their absence. Hence, the suggested link is that when herbivores are present, plants with higher direct defences receive less damage and are preferred by pollinators.

Although this mechanism is intuitive for dihydroxybenzoic acid, the opposite pattern was found for shikimic acid—where pollinators selected against this defensive compound in the presence of herbivores. Shikimic acid is most likely a common constituent of floral nectar (Hölscher et al. 2008), and like other nectar secondary compounds may be rapidly upregulated in response to herbivore attack (Adler et al. 2006; Kaczorowski et al. 2014). Thus, one explanation is that herbivore‐induced changes in this compound in nectar (or other correlated derivatives of the shikimic acid pathway) could have led to gustatory deterrence of pollinators, similar to other nectar phenolics (see Stevenson et al. 2017 and references therein). This explanation is also consistent with our finding that pollinator‐mediated selection on shikimic acid was diffuse, and disappeared in the absence of herbivores (Fig. 1). However, regardless of the underlying mechanisms, these findings nonetheless establish the capacity of pollinators to impose both positive and negative directional selection on direct defence traits of relevance to herbivores.

### SELECTION ON INDIRECT DEFENCE

Indirect defence‐related traits were in contrast selected on by both agents (Fig. 1). Herbivores selected for higher levels of dehydroascorbic acid, meaning that plants with lower levels of this carbohydrate suffered greater loss of fitness due to herbivory. The survivorship of the specialist parasitoid inhabiting the herbivore in this system is positively correlated with dehydroascorbic acid (Weber et al. 2020a). Thus, herbivore preference for plants with lower levels of this compound could relate to a natural deterrence effect of this compound (Felton and Summers 1993), or innate avoidance as a behavioral adaptation against parasitism (as per the concept of “enemy free space” [Stamp 2001]). Pollinators on the other hand positively selected for dihydroxybenzoic acid: a phenolic that—in addition to serving as a direct defence, as discussed above—is also associated with indirect defence due to its strong association with parasitism success (Weber et al. 2020a). Our finding of pollinator‐mediated selection on a trait related to indirect defence corroborates prior results indicating potential pollinator selection on volatile signals involved in tritrophic interactions and the plant's “cry for help” (Kessler and Halitschke (2009). This past work indicated potential pollinator selection on volatile signals involved in tritrophic interactions and the plant's “cry for help.” Hence in establishing the potential for pollinators to mediate selection on nonvolatile secondary metabolites, our work further illustrates the potential linkages between pollination and indirect defence. This finding is perhaps best explained as “coincidental selection,” however. Future work could aim to directly manipulate parasitism to establish whether pollinators specifically select for plants with high levels of dehydroascorbic acid.

### SELECTION ON PLANT ATTRACTION

Of the four plant attractive traits examined, significant herbivore‐ and pollinator‐mediated selection was observed only for inflorescence density (Fig. 1). Selection on this trait was both diffuse (context dependent on the other agent) and conflicting; a pattern that is likely to arise when a trait is shared as a positive host‐plant selection cue by both pollinators and herbivores (Knauer and Schiestl 2017; Ramos and Schiestl 2019). Use of this cue by the strawberry leaf beetle and another present herbivore (see Methods) appears logical given that both are florivores, and that the former is thought to occupy dense inflorescences as a means of enemy escape in its primary host plant, meadowsweet (*F. ulmaria* [L.] Maxim.) (Stenberg 2012). Shared use of this cue would explain why this trait was negatively selected by herbivores only when the influence of pollinators was controlled for, and positively selected for by pollinators when herbivores were absent. Hence, the combined effect of opposing selection by these agents was to neutralize selection overall (Fig. 1), which was also consistent with the finding that “total” section was nonsignificant (Table S4). In relation to flower frost tolerance, pollinators did not select either for or against this trait in the presence or absence of herbivores (Figure 1). Relatively stronger negative selection in their presence suggests that herbivore discrimination against frost‐damaged flowers may be more important, and was only marginally statistically insignificant. These results nonetheless highlight the need for more focused studies in this area.

## Conclusion

The findings from this study permit greater insight into the dynamics of plant‐herbivore‐pollinator systems in demonstrating how multiple selective forces may interact to shape the microevolution of traits related to pollination and herbivory. These findings therefore suggest that dynamic patterns of selection could be a common feature of these tripartite interactions in natural populations. Accordingly, the phenotypic optima of plant attractive and defence‐related traits would be expected to fluctuate across time, in accordance with changing biotic interaction strengths.

Furthermore, our study demonstrates the significant role that pollinators can play in selecting for increased direct and indirect defence‐related traits. Although the evolution of increased selfing has been proposed as one way for plants to overcome herbivore‐induced pollinator limitation (Kessler and Halitschke 2009; Penet et al. 2009; Adler et al. 2012; Johnson et al. 2015), pollinator selection for increased antiherbivore defences could offer another potentially commonplace route (Egan 2015)—and one that does not risk the disadvantages of increased inbreeding. However, ours and a previous study (Egan et al. 2016) also show that when defence‐related traits are themselves associated with pollinator limitation, then negative selection can also be expected. Together these findings highlight the complexity of selection pressures that can act on plant attractive and defence‐related traits. The predictions generated from this study may provide valuable hypotheses to test in future studies in natural populations.

## AUTHOR CONTRIBUTIONS

All authors conceived the research. JAS collected the wild plant genotypes and established the experimental common garden. PAE and AM planned and performed the experimental manipulations. PE analyzed the data and wrote the first manuscript draft. All authors provided critical input throughout the process and approved this article for publication.

## DATA ARCHIVING

https://doi.org/10.5061/dryad.1rn8pk0vn.

Associate Editor: S. Wright

## Supporting information

Ccollection of wild plant genotypes and establishment of the common garden.
**Table S1**. The 81 plant genotypes used in this study and the coordinates of their collection locality from wild populations around Uppsala County, Sweden.
**Table S2**. Selection gradient estimates for each trait, as presented graphically in Figure 1.
**Table S3**. Analyisis of variance (ANOVA) table for the selection gradient model ran in Table S2.
**Table S4**. Direction and strength of total selection on defence‐related traits and plant attractive traits in woodland strawberry (*Fragaria vesca* L.).
