# Supplementary material for: Pollinators and herbivores interactively shape selection on strawberry defence and attraction
Source: Evol Lett. 2021 Nov 14;5(6):636–43. doi: 10.1002/evl3.262 (PMC8645195; doi:10.1002/evl3.262)
Supplement: Supplementary file 1 — Ccollection of wild plant genotypes and establishment of the common garden. Table S1. The 81 plant genotypes used in this study and the coordinates of their collection locality from wild populations around Uppsala County, Sweden. Table S2. Selection gradient estimates for each trait, as presented graphically in Figure 1. Table S3. Analyisis of variance (ANOVA) table for the selection gradient model ran in Table S2. Table S4. Direction and strength of total selection on defence‐related traits and plant attractive traits in woodland strawberry (Fragaria vesca L.). [file EVL3-5-636-s001.docx]

**Supporting Information**

**Appendix 1: Collection of wild plant genotypes and establishment of the common garden**

Collection of wild plant genotypes

To harness plant genotypes that would represent a cross section of the natural genetic variation present in wild populations in Uppsala County (8207 km^2^ in area) we first divided the county into 50 equally sized squares, and then generated two random geographic positions within each square. If the two positions within a square were less than 5 km apart then two new positions were generated, replacing the two previous. For geographic coordinates see Table S1. The 100 distinct locations were visited throughout the growing season. At each location, one woodland strawberry plant was sampled. If no woodland strawberry plant was growing at the exact coordinates, then the surrounding area within a 100 m radius was carefully searched to sample the strawberry plant growing closest to the zero point. If no strawberry plants were found within the 100-meter radius then new coordinates were generated until two plants had been sampled from each square. The distances between the locations of the sampled wild strawberry genotypes varied between 7 and 40 km.

Establishment of the common garden

At SLU Ultuna campus (located in Uppsala, Sweden), the 100 sampled plant genotypes were cloned from runners for several vegetative generations. Propagated plants were transferred to the common garden in an open agricultural field in Krusenberg (N59.741°, E17.684°), 15 km south of Uppsala. The common garden consisted of 400 plants grown across four blocks spaced 1 m apart. Each block contained one clonally propagated individual of each of the 100 genotypes. Plants were randomly arranged within blocks and spaced 50 cm apart. The entire common garden was covered with MyPex® groundcover before planting in order to reduce weed densities. No irrigation or fertilizer was used. The plants were allowed to establish for one growing season prior to being used in this study.

**Table S1.** The 81 plant genotypes used in this study and the coordinates of their collection locality from wild populations around Uppsala County, Sweden. Asterisks denote the 27 genotypes analysed for defence-related traits (see Methods). These genotypes were selected across the full spectrum of variation in direct defence, as indicated by Resistance scores quantified in Weber et. al 2020a. These scores (standardized to a mean of zero) represent the outcome of genotypes screened for strawberry leaf beetle performance, with negative scores indicating a relatively poorer herbivore performance/higher genotype resistance and vice versa. Plus symbols (+) indicate genotypes for which information on indirect defence was available, based on parasitism success quantified in Figure 1 of Weber et. al 2020b.

| **Genotype** | **Latitude** | **Longitude** | **Resistance score** |
| --- | --- | --- | --- |
| 01A*^+^ | 60.58625 | 17.46078 | 0.018598016 |
| 01F | 60.60395 | 17.4834 | -0.018922053 |
| 02A* | 60.54 | 17.87583 | 0.012303137 |
| 02F* | 60.56275 | 17.8725 | 0.001365283 |
| 03A* | 60.43687 | 18.42595 | 0.010101151 |
| 03F | 60.45068 | 18.4074 | 0.001365283 |
| 04A* | 60.49272 | 17.4682 | 0.014248458 |
| 04F | 60.48858 | 17.4819 | no sample |
| 05A*^+^ | 60.43468 | 17.61243 | -0.0156571 |
| 05F | 60.44207 | 17.60162 | 0.005070305 |
| 06A* | 60.44652 | 17.77127 | -0.010705337 |
| 06F | 60.46217 | 17.79808 | 0.007605233 |
| 07F | 60.43958 | 18.01917 | 0.003288029 |
| 08A | 60.3466 | 18.47625 | 0.012711262 |
| 08F*^+^ | 60.30762 | 18.56967 | 0.017527169 |
| 09F* | 60.36547 | 17.28565 | no sample |
| 10A*^+^ | 60.3738 | 17.55967 | 0.015916149 |
| 10F | 60.38933 | 17.57033 | -0.000625967 |
| 11F | 60.35955 | 17.80812 | 0.006995097 |
| 12F*^+^ | 60.304 | 18.01518 | -0.027446501 |
| 13F | 60.21468 | 18.29258 | 0.00796838 |
| 14A | 60.23573 | 18.52 | -0.00263414 |
| 14F* | 60.23602 | 18.5572 | 0.00090935 |
| 15A | 60.30075 | 17.29102 | -0.00398043 |
| 15F | 60.33402 | 17.2302 | -0.000279259 |
| 16A | 60.27365 | 17.47525 | -0.003918525 |
| 16F | 60.25558 | 17.49052 | -0.012752451 |
| 17A* | 60.23577 | 17.68367 | -0.001435656 |
| 18A* | 60.16177 | 18.00612 | 0.012613708 |
| 18F | 60.21412 | 17.9951 | no sample |
| 19A*^+^ | 60.15827 | 18.2141 | 0.009348924 |
| 19F*^+^ | 60.19762 | 18.18657 | 0.008290844 |
| 20A | 60.09902 | 18.46335 | -0.000747265 |
| 20F*^+^ | 60.1018 | 18.34333 | -0.014939828 |
| 21A* | 60.1747 | 16.99168 | 0.003035855 |
| 21F | 60.22133 | 16.91452 | -0.006374837 |
| 22A | 60.17878 | 17.28555 | 0.005390781 |
| 22F | 60.21343 | 17.15315 | no sample |
| 23A*^+^ | 60.15597 | 17.47208 | -0.015871414 |
| 23F | 60.13857 | 17.4416 | 0.01019466 |
| 24A*^+^ | 60.0747 | 17.65225 | 0.009477936 |
| 24F | 60.13238 | 17.72247 | no sample |
| 25F | 60.04838 | 17.92115 | no sample |
| 26A | 60.0699 | 18.09048 | 0.000556034 |
| 26F | 60.01598 | 18.18887 | 0.004502213 |
| 27A | 59.99273 | 18.2636 | 0.009504466 |
| 27F | 60.03283 | 18.38825 | 0.002972768 |
| 28A | 60.0569 | 17.00835 | -0.003359373 |
| 29A | 59.99717 | 17.23442 | -0.007836966 |
| 29F | 60.01463 | 17.18172 | -0.00049472 |
| 30A | 60.00208 | 17.41463 | -0.014179984 |
| 30F | 59.98812 | 17.4263 | -0.004064443 |
| 31A | 60.00615 | 17.54802 | -0.005035639 |
| 31F | 60.01013 | 17.6086 | -0.012224085 |
| 32A | 59.95075 | 17.89972 | 0.00470751 |
| 32F | 59.97075 | 17.78635 | 0.006086931 |
| 33A | 59.94838 | 18.11503 | -0.007682431 |
| 34A | 59.91233 | 18.21488 | -0.000819949 |
| 34F*^+^ | 59.88625 | 18.2901 | -0.02694256 |
| 35A | 59.93617 | 16.88958 | 0.001902619 |
| 35F*^+^ | 59.93182 | 16.8041 | -0.019445828 |
| 36F | 59.91553 | 17.1441 | 0.000285857 |
| 37F | 59.91053 | 17.37958 | -0.006191364 |
| 38A | 59.89193 | 17.4986 | 0.001531781 |
| 38F* | 59.89763 | 17.49167 | -0.00677667 |
| 39A | 59.82633 | 17.75185 | -0.000454588 |
| 39F | 59.86815 | 17.7754 | 0.007635865 |
| 40F | 59.80065 | 18.06852 | 0.006123768 |
| 41A | 59.8421 | 16.92368 | 0.00541325 |
| 41F* | 59.8261 | 16.88697 | 0.001197148 |
| 42F | 59.8384 | 17.0513 | 0.000817691 |
| 43F* | 59.76215 | 17.34207 | -0.006788589 |
| 44F | 59.78835 | 17.54922 | 0.013607123 |
| 45A | 59.73325 | 17.7025 | -0.001105096 |
| 46A | 59.66542 | 17.06827 | -0.000994072 |
| 46F | 59.71188 | 16.93955 | 0.001932248 |
| 47F | 59.67787 | 17.29433 | 0.004764006 |
| 48F*^+^ | 59.63933 | 17.30852 | -0.01099463 |
| 49A | 59.55277 | 17.14358 | -0.003406973 |
| 50A* | 59.49877 | 17.38947 | 0.006627345 |
| 50F | 59.5279 | 17.3277 | 0.000268707 |

**Table S2.** Selection gradient estimates for each trait, as presented graphically in Figure 1. Pollinator- and herbivore-mediated selection was quantified both under control and manipulated conditions to reveal whether selection was diffuse (context-dependent on presence/absence of the other agent) and/or conflicting (exerted in opposing directions). Indicated are the treatment combinations for which differences in trait-fitness slopes were calculated to generate the selection estimates; OP (open pollination, herbivore present), OA (open pollination, herbivore absent), HP (hand pollination, herbivore present), and HA (hand pollination, herbivore absent). P-values (testing whether the differences between slopes were significantly different from zero) were adjusted by Benjamini–Hochberg correction.

| Trait | Selection type | Treatment slope difference | Selection estimate | SE | t ratio | CI lower | CI upper | P-value | P-value adjusted |
| --- | --- | --- | --- | --- | --- | --- | --- | --- | --- |
| Catechin | Herbivore-mediated (no pollen limitation) | HP - HA | -0.103 | 0.100 | -1.036 | -0.303 | 0.096 | 0.304 | 0.495 |
|  | Herbivore-mediated (pollen limitation) | OP - OA | -0.051 | 0.101 | -0.506 | -0.253 | 0.151 | 0.614 | 0.614 |
|  | Pollinator-mediated (herbivore absence) | OA - HA | -0.137 | 0.102 | -1.342 | -0.340 | 0.067 | 0.184 | 0.460 |
|  | Pollinator-mediated (herbivore presence) | OP - HP | -0.085 | 0.099 | -0.854 | -0.282 | 0.113 | 0.396 | 0.495 |
|  | Combined pollinator- & herbivore-mediated | OP - HA | -0.188 | 0.107 | -1.759 | -0.401 | 0.025 | 0.083 | 0.416 |
| Dehydroascorbic acid | Herbivore-mediated (no pollen limitation) | HP - HA | 0.200 | 0.098 | 2.038 | 0.004 | 0.396 | 0.045 | 0.076 |
|  | Herbivore-mediated (pollen limitation) | OP - OA | 0.249 | 0.096 | 2.583 | 0.057 | 0.442 | 0.012 | 0.030 |
|  | Pollinator-mediated (herbivore absence) | OA - HA | 0.093 | 0.100 | 0.924 | -0.107 | 0.292 | 0.359 | 0.359 |
|  | Pollinator-mediated (herbivore presence) | OP - HP | 0.141 | 0.094 | 1.498 | -0.047 | 0.330 | 0.139 | 0.173 |
|  | Combined pollinator- & herbivore-mediated | OP - HA | 0.342 | 0.108 | 3.175 | 0.127 | 0.557 | 0.002 | 0.011 |
| Dihydroxybenzoic acid | Herbivore-mediated (no pollen limitation) | HP - HA | -0.307 | 0.141 | -2.180 | -0.588 | -0.026 | 0.033 | 0.055 |
|  | Herbivore-mediated (pollen limitation) | OP - OA | 0.195 | 0.087 | 2.240 | 0.021 | 0.369 | 0.028 | 0.055 |
|  | Pollinator-mediated (herbivore absence) | OA - HA | -0.096 | 0.128 | -0.756 | -0.351 | 0.158 | 0.453 | 0.453 |
|  | Pollinator-mediated (herbivore presence) | OP - HP | 0.406 | 0.106 | 3.844 | 0.195 | 0.616 | 0.000 | 0.001 |
|  | Combined pollinator- & herbivore-mediated | OP - HA | 0.099 | 0.128 | 0.772 | -0.156 | 0.354 | 0.443 | 0.453 |
| Myo-inositol | Herbivore-mediated (no pollen limitation) | HP - HA | 0.072 | 0.122 | 0.590 | -0.172 | 0.316 | 0.557 | 0.897 |
|  | Herbivore-mediated (pollen limitation) | OP - OA | -0.012 | 0.091 | -0.130 | -0.194 | 0.171 | 0.897 | 0.897 |
|  | Pollinator-mediated (herbivore absence) | OA - HA | 0.099 | 0.117 | 0.846 | -0.135 | 0.334 | 0.401 | 0.897 |
|  | Pollinator-mediated (herbivore presence) | OP - HP | 0.015 | 0.097 | 0.158 | -0.179 | 0.210 | 0.875 | 0.897 |
|  | Combined pollinator- & herbivore-mediated | OP - HA | 0.087 | 0.119 | 0.736 | -0.149 | 0.324 | 0.464 | 0.897 |
| Shikimic acid | Herbivore-mediated (no pollen limitation) | HP - HA | 0.217 | 0.094 | 2.292 | 0.028 | 0.405 | 0.025 | 0.062 |
|  | Herbivore-mediated (pollen limitation) | OP - OA | -0.143 | 0.086 | -1.663 | -0.315 | 0.029 | 0.101 | 0.168 |
|  | Pollinator-mediated (herbivore absence) | OA - HA | 0.030 | 0.095 | 0.317 | -0.160 | 0.220 | 0.752 | 0.752 |
|  | Pollinator-mediated (herbivore presence) | OP - HP | -0.329 | 0.085 | -3.868 | -0.499 | -0.160 | 0.000 | 0.001 |
|  | Combined pollinator- & herbivore-mediated | OP - HA | -0.113 | 0.100 | -1.127 | -0.313 | 0.087 | 0.264 | 0.330 |
| Inflorescence density | Herbivore-mediated (no pollen limitation) | HP - HA | 0.203 | 0.365 | 0.556 | -0.526 | 0.932 | 0.580 | 0.725 |
|  | Herbivore-mediated (pollen limitation) | OP - OA | -0.391 | 0.125 | -3.121 | -0.641 | -0.141 | 0.003 | 0.013 |
|  | Pollinator-mediated (herbivore absence) | OA - HA | 0.572 | 0.224 | 2.548 | 0.124 | 1.019 | 0.013 | 0.033 |
|  | Pollinator-mediated (herbivore presence) | OP - HP | -0.022 | 0.314 | -0.071 | -0.649 | 0.605 | 0.944 | 0.944 |
|  | Combined pollinator- & herbivore-mediated | OP - HA | 0.181 | 0.213 | 0.850 | -0.243 | 0.605 | 0.398 | 0.664 |
| Total flower number | Herbivore-mediated (no pollen limitation) | HP - HA | -0.138 | 0.303 | -0.456 | -0.743 | 0.467 | 0.650 | 0.812 |
|  | Herbivore-mediated (pollen limitation) | OP - OA | 0.252 | 0.115 | 2.197 | 0.023 | 0.480 | 0.031 | 0.079 |
|  | Pollinator-mediated (herbivore absence) | OA - HA | -0.444 | 0.203 | -2.194 | -0.849 | -0.040 | 0.032 | 0.079 |
|  | Pollinator-mediated (herbivore presence) | OP - HP | -0.055 | 0.253 | -0.217 | -0.559 | 0.450 | 0.829 | 0.829 |
|  | Combined pollinator- & herbivore-mediated | OP - HA | -0.193 | 0.192 | -1.006 | -0.576 | 0.190 | 0.318 | 0.530 |
| Flower frost tolerance | Herbivore-mediated (no pollen limitation) | HP - HA | -0.333 | 0.907 | -0.367 | -2.142 | 1.476 | 0.715 | 0.715 |
|  | Herbivore-mediated (pollen limitation) | OP - OA | -2.405 | 1.013 | -2.374 | -4.426 | -0.383 | 0.020 | 0.102 |
|  | Pollinator-mediated (herbivore absence) | OA - HA | 0.367 | 0.926 | 0.396 | -1.481 | 2.214 | 0.693 | 0.715 |
|  | Pollinator-mediated (herbivore presence) | OP - HP | -1.705 | 0.995 | -1.713 | -3.691 | 0.281 | 0.091 | 0.152 |
|  | Combined pollinator- & herbivore-mediated | OP - HA | -2.038 | 1.087 | -1.875 | -4.207 | 0.131 | 0.065 | 0.152 |
| Plant size | Herbivore-mediated (no pollen limitation) | HP - HA | 0.236 | 0.252 | 0.936 | -0.267 | 0.738 | 0.352 | 0.533 |
|  | Herbivore-mediated (pollen limitation) | OP - OA | -0.225 | 0.129 | -1.743 | -0.482 | 0.033 | 0.086 | 0.215 |
|  | Pollinator-mediated (herbivore absence) | OA - HA | 0.388 | 0.211 | 1.841 | -0.033 | 0.808 | 0.070 | 0.215 |
|  | Pollinator-mediated (herbivore presence) | OP - HP | -0.072 | 0.189 | -0.383 | -0.449 | 0.304 | 0.703 | 0.703 |
|  | Combined pollinator- & herbivore-mediated | OP - HA | 0.163 | 0.204 | 0.801 | -0.244 | 0.571 | 0.426 | 0.533 |

**Table S3.** Analyisis of variance (ANOVA) table for the selection gradient model ran in Table S2.

| Parameter | Sum of squares | F-value | P-value |
| --- | --- | --- | --- |
| Catechin | 0.007 | 0.099 | 0.754 |
| Dehydroascorbic acid | 0.347 | 5.096 | 0.027 |
| Dihydroxybenzoic acid | 0.419 | 6.154 | 0.016 |
| Myo-inositol | 0.342 | 5.012 | 0.028 |
| Shikimic acid | 0.004 | 0.063 | 0.803 |
| Inflorescence density | 0.034 | 0.506 | 0.479 |
| Total flower number | 0.380 | 5.570 | 0.021 |
| Flower frost tolerance | 0.015 | 0.223 | 0.638 |
| Plant size | 0.037 | 0.546 | 0.463 |
| Pollination | 0.001 | 0.009 | 0.925 |
| Catechin:Pollination | 0.163 | 2.395 | 0.126 |
| Dehydroascorbic acid:Pollination | 0.203 | 2.972 | 0.089 |
| Dihydroxybenzoic acid:Pollination | 0.419 | 6.149 | 0.016 |
| Myo-inositol:Pollination | 0.030 | 0.437 | 0.511 |
| Shikimic acid:Pollination | 0.487 | 7.145 | 0.009 |
| Inflorescence density:Pollination | 0.282 | 4.132 | 0.046 |
| Total flower number:Pollination | 0.233 | 3.414 | 0.069 |
| Flower frost tolerance:Pollination | 0.052 | 0.768 | 0.384 |
| Plant size:Pollination | 0.061 | 0.889 | 0.349 |
| Herbivory | 0.000 | 0.006 | 0.939 |
| Catechin:Herbivory | 0.081 | 1.195 | 0.278 |
| Dehydroascorbic acid:Herbivory | 0.729 | 10.700 | 0.002 |
| Dihydroxybenzoic acid:Herbivory | 0.039 | 0.574 | 0.451 |
| Myo-inositol:Herbivory | 0.004 | 0.062 | 0.804 |
| Shikimic acid:Herbivory | 0.007 | 0.099 | 0.754 |
| Inflorescence density:Herbivory | 0.524 | 7.684 | 0.007 |
| Total flower number:Herbivory | 0.244 | 3.586 | 0.063 |
| Flower frost tolerance:Herbivory | 0.235 | 3.448 | 0.068 |
| Plant size:Herbivory | 0.086 | 1.265 | 0.265 |
| Pollination:Herbivory | 0.003 | 0.041 | 0.840 |
| Catechin:Pollination:Herbivory | 0.009 | 0.135 | 0.715 |
| Dehydroascorbic acid:Pollination:Herbivory | 0.009 | 0.126 | 0.723 |
| Dihydroxybenzoic acid:Pollination:Herbivory | 0.627 | 9.195 | 0.003 |
| Myo-inositol:Pollination:Herbivory | 0.021 | 0.303 | 0.584 |
| Shikimic acid:Pollination:Herbivory | 0.540 | 7.920 | 0.006 |
| Inflorescence density:Pollination:Herbivory | 0.161 | 2.366 | 0.129 |
| Total flower number:Pollination:Herbivory | 0.099 | 1.447 | 0.233 |
| Flower frost tolerance:Pollination:Herbivory | 0.158 | 2.323 | 0.132 |
| Plant size:Pollination:Herbivory | 0.180 | 2.648 | 0.108 |
| Residuals (df = 68) | 4.633 |  |  |

**Table S4.** Direction and strength of total selection on defence-related traits and plant attractive traits in woodland strawberry (*Fragaria vesca* L.). Presented are selection differential (S) means and their associated standard errors quantified from the control pollination-herbivore treatment combination (one plant individual per genotype). Defence-related traits were quantified for a more limited number of genotypes than plant attractive traits (see Methods). No qualitative changes in outcome were observed for plant attractive traits depending on whether the partial of full dataset were used.

|  | **Complete replication**  (n = 27 genotypes) | |  | **Expanded replication**  (n = 81 genotypes) | |
| --- | --- | --- | --- | --- | --- |
|  | Total selection  (± SE) | P-value |  | Total selection  (± SE) | P-value |
| **Defence-related traits** |  |  |  |  |  |
| Catechin | 0.017 (± 0.082) | 0.838 |  | - | - |
| Dehydroascorbic acid | 0.107 (± 0.079) | 0.190 |  | - | - |
| Dihydroxybenzoic acid | 0.193 (± 0.073) | **0.014** |  | - | - |
| Myo-inositol | -0.035 (± 0.082) | 0.673 |  | - | - |
| Shikimic acid | -0.179 (± 0.074) | **0.024** |  | - | - |
| **Plant attractive traits** |  |  |  |  |  |
| Plant size | 0.169 (± 0.075) | **0.033** |  | 0.164 (± 0.049) | **0.001** |
| Total flower no. | 0.165 (± 0.075) | **0.038** |  | 0.268 (± 0.043) | **<0.001** |
| Flower frost tolerance | -0.100 (± 0.917) | 0.914 |  | -0.035 (± 0.45) | 0.937 |
| Inflorescence density | -0.137 (± 0.078) | 0.088 |  | 0.041 (± 0.052) | 0.435 |

**References**

Weber, D., Egan, P.A., Muola, A. & Stenberg, J.A. (2020). Genetic variation in herbivore resistance within a strawberry crop wild relative (Fragaria vesca L.). *Arthropod-Plant Interactions*, 14, 31-40.

Weber, D., Egan, P.A., Muola, A., Ericson, L.E. & Stenberg, J.A. (2020a). Plant resistance does not compromise parasitoid-based biocontrol of a strawberry pest. *Scientific Reports*, 10, 1-10.
